# Arrhythmogenic right ventricular cardiomyopathy mimics: clinical impact of cardiovascular magnetic resonance

**DOI:** 10.1186/1532-429X-14-S1-P142

**Published:** 2012-02-01

**Authors:** Giovanni Quarta, Syed I Husain, Andrew Flett, Daniel Sado, William J McKenna, Antonios Pantazis, James Moon

**Affiliations:** 1 The Heart Hospital, London, UK; 2 The Institute of Cardiovascular Science, University College London, London, UK

## Summary

Several conditions detectable by CMR can mimic arrhythmogenic right ventricular cardiomyopathy. In our study, ARVC mimics found by CMR are as common as imaging ARVC criteria and clinical assessment should reflect this.

## Background

The diagnosis of arrhythmogenic right ventricular cardiomyopathy (ARVC) often relies on CMR to assess volumes, function and myocardial tissue characterization for detection of both fat and fibrosis in vivo. Clinical suspicious of ARVC often leads to CMR evaluation to refute or confirm a diagnosis. However, various diseases may mimic ARVC causing diagnostic dilemmas. We used CMR to explore the prevalence of ARVC imaging mimics in clinical practice.

## Methods

657 CMR referrals suspicious for ARVC in a single tertiary referral centre were analysed. Standardized SCMR imaging protocols for ARVC were performed. Potential ARVC mimics were grouped into: 1) displacement of the heart, 2) right ventricular overload, and 3) non ARVC-like cardiac scarring. For each, a judgment of clinical impact was made.

## Results

Twenty patients (3.0%) fulfilled imaging ARVC criteria. Thirty (4.6%) had a potential ARVC mimic, of which 25 (3.8%) were considered not epiphenomenon. These were grouped as: cardiac displacement (i.e. pectus excavatum, partial absence of the pericardium, etc) (n=17), RV overload (i.e. atrial septal defects, anomalous pulmonary venous drainage, pulmonary hypertension) (n=7) and non-ARVC like myocardial scarring (i.e. myocarditis, sarcoidosis, myocardial infarction) (n=4). One patient had two mimics; one patient had dual pathology with important mimic and ARVC. RV overload and scarring conditions were always thought clinically important whilst the importance of myocardial displacement depended on the degree of displacement from severe (partial absence of pericardium) to epiphenomenon (minor kyphoscoliosis).

## Conclusions

ARVC mimics found by CMR are as common as imaging ARVC criteria. Clinical assessment should reflect this, emphasising the assessment and/or exclusion of potential mimics in parallel with the detection of ARVC major and minor criteria.

## Funding

There are no conflict of interest to declare.

**Figure 1:**
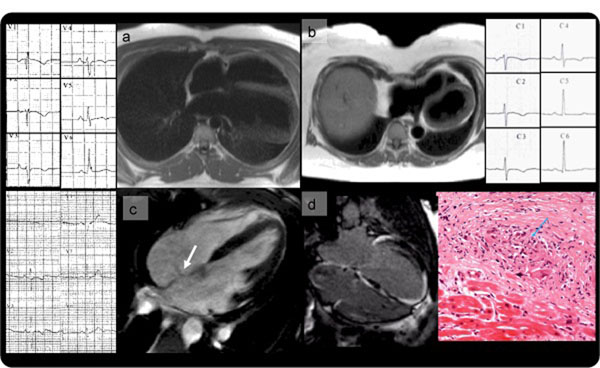
Cardiac displacement: a) Partial absence of pericardium; b) Pectus excavatum. Right ventricular overload: c) Volume loading from an atrial septal defect. Non ARVC-like myocardial scarring: d) Cardiac sarcoidosis.

